# Improving the implementation of patient safety by nursing students using nursing instructors trained in the use of quality circles

**DOI:** 10.1186/s12912-018-0318-7

**Published:** 2018-12-19

**Authors:** Linda Wieke Noviyanti, Hanny Handiyani, Dewi Gayatri

**Affiliations:** 1 Nursing Management Department, Brawijaya University, Malang, East Java 65145 Indonesia; 2 Basic Nursing Department, University of Indonesia, Depok, West Java 16424 Indonesia

**Keywords:** Patient safety, Nursing student, Quality circles, Clinical instructors

## Abstract

**Background:**

It is recognised worldwide that the skills of nursing students concerning patient safety is still not optimal. The role of clinical instructors is to instil in students the importance of patient safety. Therefore, it is important to have competent clinical instructors. Their experience can be enhanced through the application of quality circles. This study identifies the effect of quality circles on improving the safety of patients of nursing students. Patient safety is inseparable from the quality of nursing education. Existing research shows that patient safety should be emphasised at all levels of the healthcare education system. In hospitals, the ratio between nursing students and clinical instructors is disproportionately low. In Indonesia, incident data relating to patient safety involving students is not well documented, and the incidents often occur in the absence of a clinical instructor.

**Methods:**

This study used a quasi-experimental research design with pre-test and post-test non-equivalent control groups. The aim of the project was to explore the implications of the quality circle on clinical instructors by comparing the students’ knowledge, attitudes, and practices of control and intervention groups. A questionnaire will be conducted to evaluate the implementation of patient safety and the impact of the intervention. The data were statistically analysed using independent *t* tests. The intervention was the implementation of quality circles that focused on patient safety issues for the use of clinical instructors to assess and guide student nurse behaviour in regard to patient safety. The authors of this study trained the clinical instructors on how to use quality circle methods to solve nursing problems especially with relevance to the patient safety issues of students.

**Results:**

The results showed a significant increase in the behaviour of nursing students towards patient safety issues (*p* < 0.001; α = 0.05).

**Conclusions:**

The implementation of quality circles has a significant effect on patient safety. Therefore, it is recommended to implement quality circles as a problem-solving technique to optimize patient safety.

## Background

Patient safety incidents in healthcare facilities worldwide are still quite high. A previous study from the Institute of Medicine claimed that every year one million people were injured and 98,000 died as a result of medical error in the United States [1]. As many as 79.7% of the errors occurred at the time of treatment [2]. The causes of patient safety incidents involve several factors, and organizational factors are the most significant and cause 83.3% of all incidents [3]. Another factor is unsafe procedures carried out by health workers, which contributes up to 53.7% of patient safety incidents [3, 4].

Patient safety is inseparable from the quality of nursing education. Existing research shows that the introduction of patient safety should be part of the education at all levels of the healthcare system [5]. One study stated that the quality of education delivered to nursing students can significantly reduce medical errors and improve the safety culture and quality of service [6, 7]. In hospitals, the ratio between nursing students and clinical instructors is disproportionately low. In Indonesia, there are limited data on incidents related to patient safety involving students since they are not well documented and often occur without the presence of a clinical instructor [8].

Nursing students often experience real or imagined barriers associated with the clinical learning process. Students distrust their own ability which then leads to anxiety. Around 75% of the nursing students experience communication problems during the clinical learning process and 24% of them indicated that they found it difficult to endure these stressful conditions [9, 10].

The role of a clinical instructor is central to the learning process of nursing students. The clinical instructor endeavours to educate students about patient safety. This requires a good role model to embed and value the culture of safety [11]. It is therefore important to increase the expertise of the clinical instructor conducting the quality circle (QC).

The introduction of QCs is integral to improve and optimise the quality of the role of the clinical nursing instructor. Utilization of a QC can have a positive impact on the work environment. Some workers in the field agree that clinical instructors can create a positive working atmosphere and, hence, decrease the level of anxiety amongst nursing students [10] and can optimise the implementation of patient safety. QCs have never been used to guide clinical nursing students with regard to patient safety. Previous research has focused more on their effect on clinical nurses [12], job satisfaction [13, 14], and increases in motivation. An atmosphere of teamwork is strongly related with student behaviour and attitude towards patient safety. One study showed that a climate of teamwork was associated with an improved attitude among nurses towards patient safety [15]. A good team structure with good leadership and the support of clinical nursing instructors can nurture positive attitudes of student nurses toward patient safety [11, 16]. Therefore, this work aimed to study how the implementation of QCs under the control of clinical nursing instructors affected the attitude of nursing students towards patient safety.

## Methods

This study used a quasi-experimental approach, comparing the pre-test cohort of student nurses with a post-test cohort. The study was divided into two locations that involved all students at each hospital. A QC was applied to clinical instructors in the experiment group. Each clinical instructor supervises 3–8 nursing students during the implementation of plan of activation. Students were supported by a clinical instructor linked to their clinical area throughout their clinical experience. The control group only used the conventional model of clinical learning.

We selected two hospitals for this study based on a similarity of the accreditation level and number of students. To avoid a testing effect, the selected hospitals were approximately 123 km (3 h by car) apart, thereby decreasing the likelihood that students at both hospitals would communicate with each other. There was no significant difference in sociodemographic characteristics between the location of the two hospitals in terms of similarity of languages, tribes, and income. The subjects for this study were all final-year nursing students: 34 students from hospital who attended a month-long clinical placement using the QC method and 34 students from the other hospital as the control group who underwent clinical experience as usual. A non-randomized selection (based on sociodemographic characteristics) was used to determine the sample size at the two locations.

This quality circle model was delivered to the clinical instructor in the experiment group using the QC module. This module was produced by the author and involved expertise in academic and clinical nursing. Clinical instructors were first trained by the authors in QC procedures and how to produce a plan of action. Table 1 shows the detail of the QC procedure. A QC is a method of problem solving in a workplace environment using groups [13]. Once a QC has been established, it uses the following criteria in problem solving and QC development:

1. The problem has to be identified, analysed, and solved
2. The solutions have to be implemented within a designated time frame
3. Monitoring of performance has to be carried out
4. Better all-round management of QCs to encourage innovation of problem-solving methods [14]

**Table 1:** Overview of the step by step process of quality circles

	Methods
Step 1: Preparation Objectives: establishing a participatory planning group, specifying desired programme goals, making sure clinical instructors are competent to operate the quality circle (QC) process	- 1-day training programme for clinical instructors on how to operate the QC process using modules - Hold discussion sessions to brainstorm QC application for patient safety - Arrange post-test to evaluate clinical instructor knowledge after training
Step 2: Implementation Objectives: generating programme ideas, identifying problems of patient safety implementation by nursing students, stating outcomes of programme	- Identify problems faced by clinical instructors on nursing students - Make priorities from a selected problem - Arrange problem analysis using the Fishbone method - Determine and generate alternative solutions - Prepare a plan of action as a selection strategy to be used in 4 weeks
Step 3: Planning for intervention evaluation Objectives: specifying evaluation design	- Capturing the intervention effects was measured by questionnaire

The advantage of using QCs compared with other problem-solving methods, which utilize team co-operation, is that QCs are oriented to member performance and co-operation within the circle [17]. QCs are an alternative method of solving nursing problems.

The QC in this study was initiated by a voluntary gathering of a group of clinical nursing instructors interested in solving the problem of patient safety associated with nursing students. The clinical nursing instructors were first trained in the QC process to facilitate and empower them to implement and arrange a QC programme. Training is performed by an active learning method. We divided the clinical instructors into small groups to address issues related to patient safety. We brainstormed on patient safety issues faced by hospitals, especially from nursing students. The groups agreed to apply one problem according to the safety standard of patients and they selected problems regarding patient identification. The clinical instructors in the QC identified the problems encountered using Fishbone Analysis [18] to help visualise the root causes and then to find the appropriate solution. This resulted in an organized programme of measures to be taken to achieve a goal, called a plan of action (POA). The main focus of the programme was to bring about a re-socialization in the standard operating procedures such as proper patient identification, a paper-based examination to evaluate nursing student skills, and to implement a reward and punishment scheme with regard to the responses and awareness of patient safety of nursing students. It was hoped to achieve the POA in 4 weeks to improve the implementation of patient safety by nursing students.

### Data sources and measurements

The effectiveness of the intervention was measured through a pre-test and a questionnaire issued to participants approximately 4 weeks after the implementation of the intervention. Prior to applying the post-implementation survey, the authors engaged an independent team of expert reviewers, who were managers of clinical nursing instructors at different accredited teaching hospitals, to evaluate and finalize the procedure.

Data were collected using a self-administered questionnaire. Participants were asked 16 knowledge-based questions. They had to give a self-evaluation of their attitude and skills in order to assess their knowledge of patient safety. The answers were evaluated according to the three criteria: 1) “Knowledge” refers to the students’ knowledge about patient safety standards; 2) “Attitude” refers to the inward response, perception, and opinion on the implementation of patient safety standards; and 3) “Skill” refers to how skilfully patient safety standards were undertaken.

The reliability of this instrument was supported by a Cronbach’s alpha score of (*r*) > 0.76.

The ethical clearance issues was reviewed and approved by the Ethics Committee of the Nursing Science Faculty, Universitas Indonesia.

### Statistical methods

Data were analysed using the SPSS software programme. Frequency and descriptive statistics were generated for each of the variables contained in the questionnaire. Statistical analyses included univariate and bivariate analysis.

Univariate analysis was conducted to describe the characteristics of each of the variables studied. Numerical data such as age, length of clinical education, patient safety knowledge, student attitudes, and student actions regarding patient safety are presented as mean, median, and standard deviation (SD) with 95% confidence interval. Categorical data such as sex, educational level, exposure about patient safety, hospital orientation, and the status of the institution from which the student comes were presented as a frequency distribution and proportion.

Bivariate analysis was conducted to find out the relationship between dependent and independent variables. The purpose of this research analysis is to show the significant difference between two groups—the intervention and the control group. This research was conducted with an equality test in the intervention group and control group before the intervention was performed. The equality test for the age variable, professional life, knowledge, attitudes, and safety measures of patients which are numerical data is made using an independent test sample *t* test. Variables that use categorical data were tested using Chi Square test analysis.

The bivariate analysis was performed to: 1) compare mean scores between pre- and post-tests in the intervention and control groups using dependent sample *t* tests (a *p* value < 0.05 from the *t* test would imply that there is significant difference between the pre-test and post-test both in the control and intervention group); and 2) compare the mean difference between scores of pre- and post-test in the intervention and control groups using independent sample *t* tests (with a significant mean difference between control and intervention group described by a *p* value < 0.05).

## Results

A total of 68 students (response rate = 85%) participated in the study; students who did not participate for different reasons (absent on the day of tool administration, or refusal to participate) were homogeneously distributed, with those with just bachelor degrees involved. A total of 68 students were attending first-year clinical education, and the length of clinical education undertaken by students in the intervention group was 9.53 ± 0.50 months (mean ± SD 0.50) compared with 4.97 ± 1.29 months for the control group. As reported in Table 2, the majority of students were female (76.5%), and their mean age was 22.91 ± 0.86 years in the intervention group and 22.82 ± 0.83 years in the control group**.** In both groups, 29 students (85.3%) had previous exposure to patient safety information, while five (14.7%) in both groups reported never having received information about patient safety. All students (100%) in the control group received patient safety orientation from their hospital while six students (17.7%) in the intervention group had not received orientation from their hospital.

**Table 2:** Demographics

Variable	Intervention group	Control group	Total
Age (years), mean ± SD	22.91 ± 0.86	22.82 ± 0.83	**–**
Clinical education (months), mean ± SD	9.53 ± 0.50	4.97 ± 1.29	**–**
Sex			
Male	8 (23.5%)	8 (23.5%)	16 (23.5%)
Female	26 (76.5%)	26 (76.5%)	52 (76.5%)
Total	34 (100%)	34 (100%)	68 (100%)
Education background			
Bachelor	34 (100%)	34 (100%)	68 (100%)
Total	34 (100%)	34 (100%)	68 (100%)
Did you have exposure to patient safety?			
Received	29 (85.3%)	29 (85.3%)	58 (85.3%)
Never received	5 (14.7%)	5 (14.7%)	10 (14.7%)
Total	34 (100%)	34 (100%)	68 (100%)
Hospital orientation			
Yes	28 (82.3%)	34 (100%)	62 (91.2%)
No	6 (17.7%)	0 (0%)	6 (8.8%)
Total	34 (100%)	34 (100%)	68 (100%)
Institution status of student			
Public	20 (58.8%)	0 (0%)	20 (29.4%)
Private	14 (41.2%)	34 (100%)	48 (70.6%)
Total	34 (100%)	34 (100%)	68 (100%)

Values are shown as *n* (%) unless otherwise stated

*SD* standard deviation

Table 3 shows the pre-test and post-test mean scores of “Knowledge”, “Attitude”, and “Skills” of the intervention group and the control group. “Knowledge” increased in both groups but with large SDs. The changes were not significant (*p* = 0.002; α = 0.05).

**Table 3:** Mean score of the implementation of the safety of patients in the intervention group and the control group based on the pre-test and post-test

Variables	*n*	Mean	SD	*p*
Knowledge				
Intervention group				
Pre-test	34	71.88	11.32	0.002
Post-test	34	78.12	10.15	
Control group				
Pre-test	34	57.06	6.480	< 0.001^a^
Post-test	34	64.59	8.76	
Attitude				
Intervention group				
Pre-test	34	108.47	7.15	0.792
Post-test	34	109.29	18.01	
Control group				
Pre-test	34	104.56	7.18	0.797
Post-test	34	104.15	7.47	
Skill				
Intervention group				
Pre-test	34	115.79	13.18	< 0.001^a^
Post-test	34	125.71	12.93	
Control group				
Pre-test	34	118	10.71	< 0.001^a^
Post-test	34	105.06	18.10	

*SD* standard deviation

^a^α = 0.05

The post test score for patient safety attitude shows insignificant change from the pre-test scores.

Skills increased significantly in the intervention group but declined insignificantly in the control group. Further test analysis showed the skill levels had increase in the control group as well.

Table 4 compares the “Knowledge”, “Attitude” and “Skill” parameters between the intervention and control groups with regard to patient safety. Surprisingly the mean “Knowledge” score was lower in the intervention group but was statistically insignificant.

**Table 4:** The mean difference of patient safety implementation between intervention and control groups based on the results of the pre-test and post-test

Variables	*n*	Mean of differences	SD	*t*	*p*
Knowledge					
Intervention group	34	6.24	11.01	−0.50	0.616
Control group	34	7.53	10.12		
Attitude					
Intervention group	34	0.82	18.06	0.35	0.724
Control group	34	−0.41	9.25		
Skill					
Intervention group	34	9.91	15.26	5.65	< 0.001^a^
Control group	34	−12.94	17.96		

*SD* standard deviation

^a^α = 0.05

The results of the analysis of patient safety Attitude showed no significant difference between the two groups.

Analysis of the “Skills” statistics showed that the use of QC by nursing instructors improved the patient safety skills of nursing students significantly.

## Discussion

Analysis of the statistics showed that there was no significant increase in the attitude of student nurses to patient safety after the implementation of the QCs. However, in the main, the mean scores of the parameters measured increased in the intervention group. A previous study found that inexperience and distraction were the leading factors contributing to student errors [19]. Other research findings on medication errors and associated system factors have not significantly informed nursing practice. In addition, it shows there is a lack of active partnerships between nursing education and hospital quality assurance systems [20]. Without proper regard to patient safety, the health and welfare of a patient can suffer. Death can even occur in rare instances. The wrong patient can be given drugs intended for someone else, wrong doses, and inadequate supervision and monitoring of the patient leading to adverse events.

The significant increase in “Knowledge” in the control group from our analysis is dominated by certain factors, among them the process of clinical guidance. The control hospital has better clinical counselling arrangements than the intervention group hospital. Furthermore, the nurse selected as the clinic supervisor at the control hospital is not concentrated as head of the room. In addition, clinical instructors follow an “office”-type schedule of shifts rather than just the morning service, meaning the supervision process for students other than in the morning can also run well. This can affect the implementation of patient safety since the role of clinical instructors can run optimally. The results of research indicate that the benefits gained by students during the optimal clinical counselling process is increased knowledge [21].

Our analysis from a management function perspective is that the change of “knowledge” in the control group and the intervention group is not separate from the management function provided by clinical instructors [10]. Directive functions had the purpose of setting, directing, and optimizing resources to achieve organizational goals [22]. One of the activities of a directive function is supervision activities [19]. The optimal implementation of patient safety by students is inseparable from the role of clinical instructors since the role of an effective clinical instructor is related to improving the autonomous ability and psychological mental readiness of the student undergoing the transition process to a professional nurse [23, 24]. Clinical instructors who are able to provide motivation and supervise the students contribute to the optimal implementation of patient safety [10].

We believe that QCs influenced the work environment in the intervention group by subjecting them to more practice regarding patient safety. This is supported by the significant improvement in patient safety skills in the intervention group. We believe that this results from the implementation of QCs which create a positive work environment and a sense of belonging to the organization [25].

Clinical practice settings can positively contribute to patient safety, as shown by a study that found good clinical practice environments affected patient outcomes [7, 26]. Patient outcomes in this study were associated with the implementation of patient safety procedures. Implementation of QCs can create a positive environment for nursing students to gain confidence and develop a positive attitude to patient safety procedures. Regarding our analysis, the cause of a significant increase in the intervention group was due to the application of QC clinical instructors. Application of a POA from QC meetings led to awareness of the importance of supervision by clinical instructors. The quality of supervision from the clinical counsellor influences the professional ability of nursing students. This is in line with the results of a study that supervision can improve the professional values associated with patient safety [23].

There was a significant increase in the skills of nursing students regarding patient safety. This was measured by the difference between pre-test and post-test results. QC implementation for clinical nursing instructors positively affected the patient safety skills of students, but did not show significant improvements in their knowledge or attitude. This may be due to many factors, such as the attributes of the nursing instructor or of the nursing student, or both.

It was found that the consistency and commitment of the clinical instructors running the QC programmes was low. The success of a programme like this depends, to a large extent, on the ability of QC members to analyse problems [27]. We observed that technical factors led to the non-optimum implementation of QCs, the main factor one being the inadequate preparation of the QCs. The clinical instructors, despite having completed a QC training session, were still not able to incorporate their findings from the Fishbone Analysis into their POAs.

The improvement in patient safety resulted from the clinical instructors’ POA which focussed on the students’ safety skills. This is in line with other research that shows QC programmes focussing on improving psychomotor skills have an impact, but do not improve peoples’ attitude [10]. This finding justifies the implementation of QCs because they can significantly affect the patient safety skills of nursing students.

Discussions were held with nursing management to obtain their perspective and to develop their understanding of, and involvement with, QCs. They felt that the use of QCs could not be differentiated from optimal management procedures. However, the authors communicated that QCs are a problem-solving technique that integrates the functions of management and their responsibilities. The responsibility of management is to ensure that the learning process of nursing students should be guided by competent nurse instructors. It was pointed out that QC methods are useful for human resource management because they contain elements of management participation [13]. Management staff involvement impacts job satisfaction and helps assign staff with optimal capabilities to the organization. This is important so that the implementation of patient safety is initiated properly.

The hospital management decided which experienced nurses were to be assigned to the QCs. Often, only those with diploma qualifications, rather than more highly qualified nurses with bachelor degrees, were assigned. This may help explain why the POAs were not implemented properly and why there was no significant increase in the knowledge and attitudes of patient safety.

We found that management did not supervise the QCs well. Management should maintain quality through evaluating and monitoring activities [28, 29]. Ideally, QC intervention should have been performed for a period of at least 3 months [30] to obtain the full support of management. In this study, the authors acted as facilitators to ensure optimal implementation of the QC programmes. However, it was felt that the time allocated for the implementation of the QCs was too short and that programme supervision was not performed adequately. This affected the success of the QCs in optimizing patient safety.

### Limitations

Respondents originated from one region only, which is a potential limitation to generalizing our results to the national population of nursing students. Therefore, an investigation regarding to what extent population characteristics of samples differ from the national population of nursing students is needed.

The study also has limitations related to technical research and the length of intervention. The authors developed the instrument based on six patient safety goals. This study used five patient safety goals to allow for adjustment with the study respondents, and therefore required continuous evaluation to test the consistency of the use of the instrument for the next study. The technical implementation of the research still has limitations due to the non-ideal implementation of the application of QCs. Scheduling an evaluation which should be done twice, could only be done once at the end of the session. Implementation of a QC programme ideally requires 6 months from the planning stage up to the evaluation, but in this study it was implemented in just a month. Furthermore, we were also not yet supposed to be getting QC managerial support from the hospital.

## Conclusions

The application of QCs for clinical nursing instructors significantly improved the patient safety implementation of nursing students, especially skills. Suggestions and recommendations from this study are that the hospital should use the results of this research to formulate policies and procedures for coaching clinics to improve patient safety by nursing students. The authors advise the use of a combination of other interventions alongside QCs to improve the quality of hospital services. The students’ attributes could not be controlled by the authors as the students were allocated to the study groups by the hospital. In addition, we feel that the type of institution where students were trained may also affect the performance and the application of the results of these QCs.

A follow up study should be performed by controlling characteristics of the students such as the length of professional service, hospital orientation, and type of institution the students studied at, whether private or government, especially if using undergraduate nursing students as samples.

## Abbreviations

POA: Plan of action
QC: Quality circle
SD: Standard deviation
